# Metabolically active, non-nitrogen fixing, *Trichodesmium* in UK coastal waters during winter

**DOI:** 10.1093/plankt/fbv123

**Published:** 2016-05-30

**Authors:** Andrew P. Rees, Karen Tait, Claire E. Widdicombe, Graham D. Quartly, Andrea J. McEvoy, Lisa Al-Moosawi

**Affiliations:** Plymouth Marine Laboratory, Prospect Place, The Hoe PL1 3DH, UK

**Keywords:** *Trichodesmium*, English Channel, Western Channel Observatory, Winter, ^13^C, ^15^N, PCR

## Abstract

*Trichodesmium*, a colonial cyanobacterium typically associated with tropical waters, was observed between January and April 2014 in the western English Channel. Sequencing of the heterocyst differentiation (*hetR*) and 16S rRNA genes placed this community within the Clade IV *Trichodesmium*, an understudied clade previously found only in low numbers in warmer waters*.* Nitrogen fixation was not detected although measurable rates of nitrate uptake and carbon fixation were observed. *Trichodesmium* RuBisCO transcript abundance relative to gene abundance suggests the potential for viable and potentially active *Trichodesmium* carbon fixation. Observations of *Trichodesmium* when coupled with a numerical advection model indicate that *Trichodesmium* communities can remain viable for >3.5 months at temperatures lower than previously expected. The results suggest that Clade IV *Trichodesmium* occupies a different niche to other *Trichodesmium* species, and is a cold- or low-light-adapted variant.

*Trichodesmium* is a filamentous, non-heterocystous cyanobacterium that contributes significantly to the biological fixation of nitrogen in tropical and sub-tropical waters (Carpenter, 1983; Bergman *et al*., 2012) and may contribute up to 47% of primary production in the tropical North Atlantic (Carpenter *et al*., 2004). Diazotrophic activity by this genus is thought to contribute between 60 and 80 Tg N annually (Bergman *et al*., 2012) to a global marine N fixation budget of 100–200 Tg N per year (Karl *et al*., 2002), making this an important group of organisms for the productivity and functioning of the warm, oligotrophic environment within which their distribution is largely limited. Although the occurrence of metabolically active *Trichodesmium* appears to be mostly restricted to water temperatures of >20°C, Carpenter (Carpenter, 1983) introduces a number of accounts of *Trichodesmium* appearing in colder conditions. McCarthy and Carpenter (McCarthy and Carpenter, 1979) reported active populations of *Trichodesmium* at 18.3°C in the North Atlantic, and Farran (1932) reported collecting *Trichodesmium* in 75% of plankton samples from the south of Ireland between October and March in temperatures of 9–12°C. Indications are that *Trichodesmium* occurs episodically in northern latitudes of the Atlantic following passive transport along ocean currents (Laroche and Breitbarth, 2005 and references therein) and that cells are capable of surviving for several weeks at temperatures at least as low as 17°C (Breitbarth *et al*., 2007) and possibly even in Arctic environments (Díez *et al*., 2012).

This paper describes observations made of the persistent occurrence of *Trichodesmium* at stations L4 and E1 of the Western Channel Observatory (WCO, http://www.westernchannelobservatory.org.uk/) over 12 weeks between January and April 2014. The winter of 2013 into 2014 for the UK, western English Channel and the greater north east Atlantic was characterized by persistent periods of low pressure which resulted in consistently strong winds originating in the south and west and prolonged periods of heavy rainfall (http://www.metoffice.gov.uk/climate/uk/summaries/2014). Seawater temperatures at L4 decreased from 12.2°C in early December to 8.9°C by 5 March and then increased up to 10.2°C by 9 April, which is typical for this region (Smyth *et al*., 2010). Nitrate concentrations were generally typical of winter conditions: 6.4 µmol L^−1^ on 2 December 2013, 7.9 µmol L^−1^ on 18 February 2014 and 8.6 µmol L^−1^ on 9 April (see http://www.westernchannelobservatory.org.uk/l4_nutrients.php for the full dataset). Mean and median concentrations between 2 December 2013 and 9 April 2014 were 9.2 and 8.5 µmol L^−1^, respectively.

Colonies of suspected *Trichodesmium* were first recorded in zooplankton samples collected at station L4 on 14 January 2014. These samples are routinely collected as part of the WCO monitoring programme with standardized protocols to provide a semi-quantitative assessment of plankton abundance or biomass. Full details are provided elsewhere (UNESCO, 1968, p. 153–157; Eloire *et al*., 2010). The sampling protocol involves vertical hauls of a WP2 net (57-cm diameter, 200-µm mesh size) from 50 m (bottom depth ∼55 m) to the surface at a winch speed of ∼20 cm s^−1^. Samples were kept in cool, dark conditions, returned to the laboratory and processed under ambient conditions. A number of colonies were either collected directly onto 25-mm GF/F filters (Fig. 1a) or gently removed from the concentrated net samples using forceps and examined in 3-mL aliquots of filtered seawater using a Leica DMI4000B light microscope at magnifications between ×4 and ×20 (Fig. 1b and c). *Trichodesmium* was observed on 11 of 13 sampling occasions between 14 January and 9 April 2014. Colony abundance was enumerated from the WP2 net hauls on three occasions, with counts of 663, 194 and 17 colonies m^−3^ estimated on 10 February, 18 February and 5 March, respectively, using light microscopy. As a flow-meter is not fitted to WP2 nets, the volume of water filtered is estimated from the product of net opening area (0.25 m^2^) × depth sampled (50 m) × sampling efficiency coefficient (0.95).

**Fig. 1.**
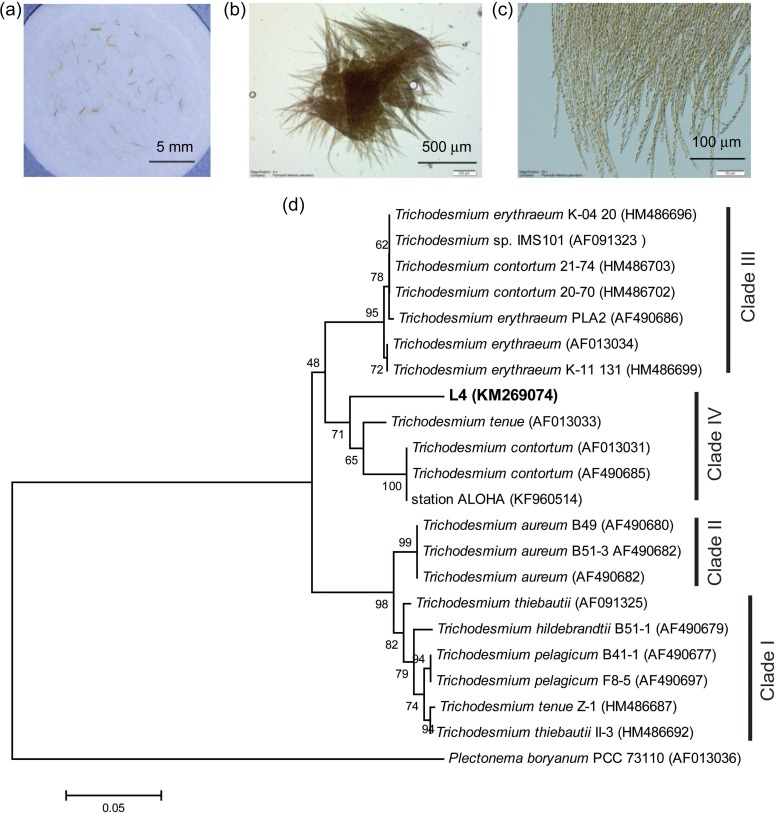
*Trichodesmium* colonies collected at station L4 in the western English Channel, January 2014 at (**a**) ×1, (**b**) ×4 and (**c**) ×20 magnification, respectively. The phylogenetic tree (**d**) is based on *het R* gene sequences with maximum likelihood and was bootstrapped 1000 times. The L4 sequences are compared with *Trichodesmium* sequences from Genbank. Shown are the four clades identified by Hynes *et al*. (Hynes *et al*., 2012).*Oscillatoria sancta* PCC7515 was used as an outgroup to root the tree. Numbers at nodes reflect percent bootstrap consensus >50%.

In order to identify the population, DNA was extracted from colonies from three separate occasions (14 January, 29 January and 10 February) which had been collected onto 25-mm, 0.8-µm polycarbonate filters which were frozen at −80°C. DNA was extracted from the filaments using the method of Hoffman and Winston (Hoffman and Winston, 1987) and amplified using three different PCR primer pairs previously used to identify *Trichodesmium* sp. (Hynes *et al*., 2012) (molecular methods can be found in Supplementary Information). Sequences from each of the three different genes studied had >99% sequence similarity, suggesting that only one species was present. Hynes *et al*. (Hynes *et al*., 2012) identified four clades of *Trichodesmium* from cultured representatives and field samples. The filaments found at Station L4 were most closely related to *Trichodesmium*, with the 16S rRNA and *hetR* (Fig. 1d) gene sequences both indicating the colonies grouped with Clade IV. Clade IV *hetR* sequences were derived from *Trichodesmium* colonies collected from the Caribbean Sea (*T. contortum* AF013031), close to the equator in the Atlantic Ocean (*T. tenue* AF013033, Janson *et al*., 1999), and also station ALOHA (KF960514) in the Pacific. The *Trichodesmium* colonies found at L4 had 97% *hetR* sequence similarity to Clade IV *Trichodesmium* previously isolated from the Caribbean Sea (Janson *et al*., 1999). Similarly, the L4 16S rRNA gene sequence had 99% sequence similarity to the Clade IV *Trichodesmium*. Other studies that have included descriptions of Clade IV *Trichodesmium* are limited. However, one study of the diversity of *Trichodesmium* at station ALOHA at 10 m depth over a 2-year period demonstrated only 2% of *hetR* gene sequences grouped with Clade IV (Gradoville *et al*., 2014). It is possible that Clade IV may be more suited to cooler waters than those associated with tropical regions. Alternatively, this group may be adapted to lower light conditions. The Clade IV *T. tenue* identified by Janson *et al*. (Janson *et al*., 1999) close to the equator in the Atlantic Ocean was isolated from a depth of 75 m, and it was noted that this type was not found at depths shallower than 25 m. Together, this suggests that Clade IV *Trichodesmium* may occupy a different niche than other *Trichodesmium* species.

Following the observation of *Trichodesmium-*like cyanobacteria in low-temperature environments (Carpenter, 1983; LaRoche and Breitbarth, 2005; Díez *et al*., 2012), the question arises as to whether these populations are growing, dormant or dead. Two approaches were taken to investigate their activity. One method involved using stable isotopes to follow the incorporation of N_2_, NO_3_^−^ and CO_2_ into colonies. As it is possible that this approach may be influenced by the complex consortia of phytoplankton and epibiotic bacteria often associated with *Trichodesmium* sp. colonies (Sheridan *et al*., 2002; Hmelo *et al*., 2012), molecular techniques were also used to investigate RuBisCO activity to provide a more direct insight into photosynthetic activity.

^15^N and ^13^C isotopes were used during a single investigation which was performed on colonies collected on 30 January. Colonies were picked using forceps and a binocular microscope, rinsed in filtered sea water and then distributed into 3×125 mL serum bottles where they were re-suspended in 0.2-µm filtered seawater collected immediately before plankton net hauls. A single bottle containing 60 colonies was filled and crimp sealed with a Teflon backed butyl septa. A second bottle containing 60 colonies was amended with 620 µmol L^−1^ of ^13^C-labelled bicarbonate to determine carbon fixation. This was capped as previously and then further amended with the addition of 1 mL of 98 atom% ^15^N_2_ gas (Cambridge Isotope Laboratories, Inc., LOT I1-11785A) to determine nitrogen fixation (e.g. Rees *et al*., 2009). A final bottle containing 30 colonies was amended with additions of 620 µmol L^−1^ of ^13^C bicarbonate and 0.4 µmol L^−1^ of ^15^N-labelled nitrate to determine carbon fixation and nitrate uptake (e.g. Bury *et al*., 2012). All three bottles were transferred to a laboratory incubator for 24 h and maintained at 10°C for 12 h in the light (∼100 µE m^−2^ s^−1^) and 12 h in the dark. At the end of the incubation, they were filtered onto 25-mm GF/F filters and dried for 12 h at 50°C. ^13^C and ^15^N atom% was then determined using stable isotope mass spectrometry (SERCON 20:20 and GSL); precision of analysis was 0.26 and 0.03% coefficient of variation for ^15^N and ^13^C, respectively.

Relative to the control treatment, which received no additions, it is not surprising that there was no enrichment of ^15^N in cells contained within the bottle amended with ^15^N-N_2_. This supports the accepted wisdom that *Trichodesmium* does not fix nitrogen under low-temperature and high-nutrient conditions (Carpenter, 1983; Capone *et al*., 1997; Holl and Montoya, 2005; Breitbarth *et al*., 2007). With the major caveat that there was likely to be a microbial community associated with incubated *Trichodesmium* (Sheridan *et al*., 2002; Hmelo *et al*., 2012), indications are that the populations of the Clade IV *Trichodesmium* were capable of the fixation of carbon and uptake of NO_3_^−^ (Fig. 2). Enrichments of 0.0088 atom% ^15^N and 0.0604 and 0.0742 atom% ^13^C were observed which equate to rates in the order of 0.05 nmol NO_3_^−^ colony^−1^ day^−1^ and 0.42 nmol C colony^−1^ day^−1^, and a ratio of carbon fixation:nitrate uptake of 8:1. With other nitrogen sources available including NH_4_^+^ (mean concentration of 0.52 µmol L^−1^ for December 2013 to February 2014), it is not unreasonable to assume that C:N uptake rates were in balance relative to Redfield stoichiometry. Whether attributable solely to *Trichodesmium* or to an associated microbial community, these rates do not present a significant contribution to the productivity of the western English Channel. The observed C fixation was an order of magnitude lower than the lowest rates of primary production reported by Carpenter *et al*. (Carpenter *et al*., 2004) for the tropical North Atlantic, and based on the maximum observed abundance of 663 colonies m^−3^ contributes <0.03% of wintertime primary production of 12.7 (±7.1) mgC m^−3^day^−1^ reported by Barnes *et al*. (Barnes *et al*., 2015).

**Fig. 2.**
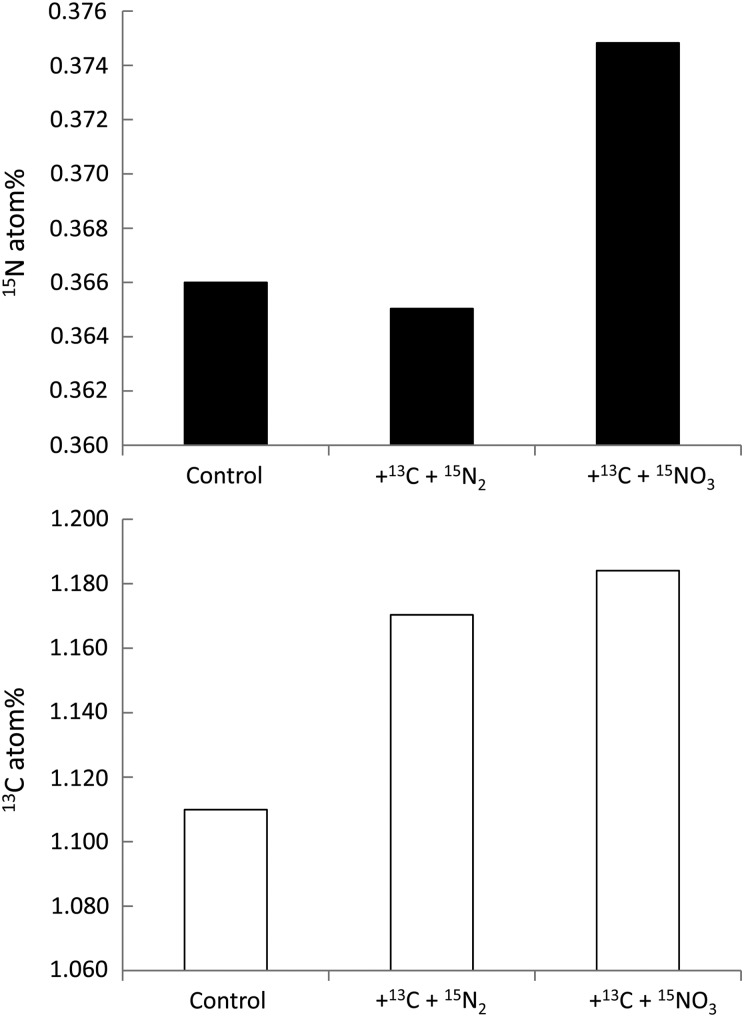
Enrichment of *Trichodesmium* colonies in ^15^N (upper panel) and ^13^C (lower panel) following incubation with dual additions of ^13^C bicarbonate plus ^15^N – N_2_, and ^13^C bicarbonate plus ^15^N – NO_3_^−^. Control and ^13^C+^15^N_2_ had 60 *Trichodesmium* colonies, while ^13^C +^15^NO_3_ had 30 *Trichodesmium* colonies.

To confirm that the *Trichodesmium* were able to fix carbon, quantitative PCR (qPCR) was used to measure the abundance of transcripts of the large subunit of RuBisCO (*rbcL*) relative to gene numbers. Colonies collected on two occasions (29 January and 10 February) were subjected to a combined DNA and RNA extraction procedure, as described in Supplementary Information. Primers specific to the *rbcL* gene of *T. erythraeum* were designed, PCR amplified from cDNA and DNA and the resultant PCR product was cloned and sequenced. Eight clones were sequenced from each time-point: all contained the same gene sequence which had 92 and 99% identity to the *T. erythraeum rbcL* gene and protein sequence, respectively (accession number KU221396). Quantification of *rbcL* mRNA and DNA indicated the *rbcL* mRNA:DNA ratio to be 25.8 (±10.04 SD; *n* = 2). Measurements of both *rbcL* gene and transcript abundance from other studies are rare. For diatom species in the Bering Sea, diatom-specific *rbcL* transcripts relative to genes numbered >1000 (Endo *et al*., 2015). A recent study of the impact of CO_2_ and temperature on *Trichodesmium* gene expression reported expression of *rbcL* relative to 16S rRNA abundance, in the range of 10^0^–10^1^ copies of *rbcL*:16S rRNA (Hewson *et al*., 2009). Although the expression levels of *rbcL* measured in this current study were low, the presence of *rbcL* mRNA does indicate that the *Trichodesmium* sp. found at L4 were capable of fixing carbon.

*Trichodesmium* spp. are generally associated with oligotrophic sub-tropical and tropical waters with a temperature range of 20–30°C (Breitbarth *et al*., 2007). To assess the transport of this population to the WCO, we have considered satellite observations of surface temperatures and currents (Fig. 3) to provide some constraints as to how long these trichomes may have experienced waters cooler than 20°C. The seasonal variations in sea surface temperature in the northern hemisphere peak in September, with the isotherms at their northernmost extent. The mean currents in the region, including the eastward-flowing North Atlantic Current and the poleward-flowing European Slope Current in the Bay of Biscay (Xu *et al*., 2015) provide some transport to the WCO site. To include the action of eddies, an advective model was used to transport individual particles from the realm bounded by the 20 and 21°C isotherms in September 2013. The numerical model uses horizontal surface current velocities produced at daily resolution by the SURCOUF component of GlobCurrent (www.globcurrent.org). The current field includes both the geostrophic component, calculated from a number of altimeters, and the surface Ekman current calculated from the vector wind field (Rio *et al*., 2014). The inclusion of the eddy field increases the dispersion of particles compared with simply using a set of mean currents, but even so requires more than the 3.5 months until the start of 2014 to explain the presence of viable *Trichodesmium* at the WCO (Fig. 3).

**Fig. 3.**
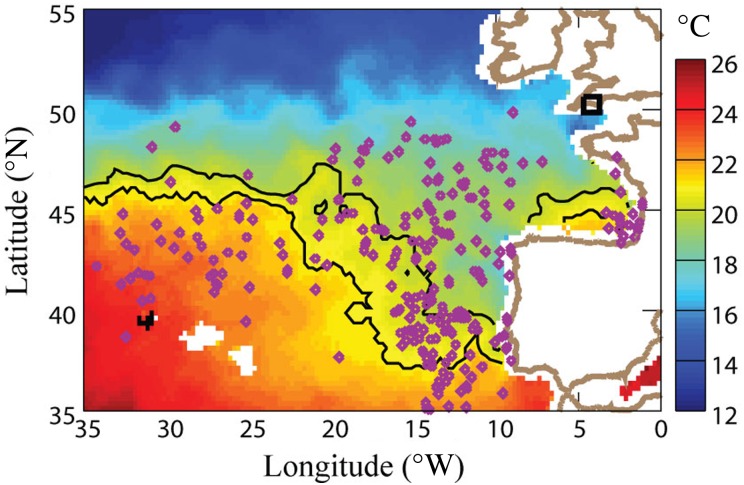
Sea surface temperature (SST) from AMSR-2 for September 2013, with the 20 and 21°C isotherms highlighted in black. The region between these isotherms was seeded with virtual drifters and daily geostrophic and Ekman currents applied until 1 January 2014, resulting in the distribution of diamond markers. The open black square represents the location of the Western Channel Observatory.

We also examined the SST distribution for September during other years to note whether 2013 was in any way different. In fact, although the 20°C isotherm at 20°W was at its furthest north in 2013, for regions east of 15°W, the temperatures were cooler than normal, with that isotherm displaced southwards, which indicates that a contribution via the European Slope Current (Xu *et al*., 2015) can also be discounted.

## 

We find that RuBisCO transcript abundance relative to gene abundance suggests the potential for viable and potentially active Clade IV *Trichodesmium* in the western English Channel. Indications are that this group can remain viable for many months under environmental conditions previously considered to preclude its activity. Although the rates of carbon fixation and nitrate uptake observed do not indicate a major role for this community in the biogeochemical cycling of these waters, their occurrence is ecologically unprecedented and informs on the transport and distribution of this globally significant cyanobacterium.

## FUNDING

This work was supported by the UK Natural Environment Research Council (NERC) through National Capability funding of the Western Channel Observatory (WCO). The authors acknowledge the sustained contribution made by the crew of the RV Plymouth Quest. Funding to pay the Open Access publication charges for this article was provided by the UK Natural Environment Research Council.

## Supplementary Material

Supplementary Data

## References

[FBV123C1] Barnes M., Tilstone G. H., Suggett D., Widdicombe C., Bruun J., Martinez-Vicente V., Smyth T. J. (2015) Temporal variability in total, micro- and nano-phytoplankton primary production at a coastal site in the western English Channel. Prog. Oceanogr. (online). doi:10.1016/j.pocean.2015.04.017.

[FBV123C2] Bergman B. B., Sandh G., Lin S., Larsson J., Carpenter E. J. (2012) *Trichodesmium*—a widespread marine cyanobacterium with unusual nitrogen fixation properties. FEMS Microbiol. Rev., 37, 286–302. 22928644 10.1111/j.1574-6976.2012.00352.x PMC3655545

[FBV123C3] Breitbarth E., Oschlies A., Laroche J. (2007) Physiological constraints on the global distribution of *Trichodesmium*—effect of temperature on diazotrophy. Biogeosciences, 4, 53–61.

[FBV123C4] Bury S. J., Zeldis J. R., Nodder S. D., Gall M. (2012) Regenerated primary production dominates in a periodically upwelling shelf ecosystem, northeast New Zealand . Continental Shelf Res., 32, 1–21.

[FBV123C5] Capone D. G., Zehr J. P., Paerl H. W., Bergman B., Carpenter E. J. (1997) *Trichodesmium*, a globally significant marine cyanobacterium. Science, 276, 1221–1229.

[FBV123C6] Carpenter E. J. (1983) Chapter 3—Nitrogen fixation by marine Oscillatoria (Trichodesmium) in the world's oceans. In Carpenter E. J., Capone D. G. (eds) Nitrogen in the Marine Environment. Academic Press, San Diego, pp. 65–103.

[FBV123C7] Carpenter E. J., Subramaniam A., Capone D. G. (2004) Biomass and primary productivity of the cyanobacterium *Trichodesmium* spp. in the tropical N Atlantic ocean. Deep Sea Res. Part I, 51, 173–203.

[FBV123C8] Díez B., Bergman B., Pedrós-Alió C., Antó M., Snoeijs P. (2012) High cyanobacterial nifH gene diversity in Arctic seawater and sea ice brine. Environ. Microbiol. Rep., 4, 360–366. 23760800 10.1111/j.1758-2229.2012.00343.x

[FBV123C9] Eloire D., Somerfield P. J., Conway D. V. P., Halsband-Lenk C., Harris R. (2010) Temporal variability and community composition of zooplankton at station L4 in the Western Channel: 20 years of sampling. J. Plankton Res., 32, 657–679.

[FBV123C10] Endo H., Sugie K., Yoshimura T., Suzuki K. (2015) Effects of CO_2_ and iron availability on *rbcL* gene expression in Bering Sea diatoms. Biogeosciences, 12, 2247–2259.

[FBV123C27] Farran G. P. (1932) The occurrence of *Trichodesmium thiebautii* off the south coast of Ireland. Rapp. P.-V. Reun. Cons. Perm. Int. Explor. Mer., 77, 60–64.

[FBV123C11] Gradoville M. R., White A. E., Boettjer D., Church M. J., Letelier R. M. (2014) Diversity trumps acidification: lack of evidence for carbon dioxide enhancement of *Trichodesmium* community nitrogen or carbon fixation at Station ALOHA. Limnol. Oceanogr., 59, 645–659.

[FBV123C12] Hewson I., Poretsky R. S., Dyhrman S. T., Zielinski B., White A. E., Tripp H. J., Montoya J. P., Zehr J. P. (2009) Microbial community gene expression within colonies of the diazotroph, *Trichodesmium*, from the Southwest Pacific Ocean. ISME J., 3, 1286–1300. 19571897 10.1038/ismej.2009.75

[FBV123C13] Hmelo L. R., Van Mooy B. A. S., Mincer T. J. (2012) Characterization of bacterial epibionts on the cyanobacterium *Trichodesmium*. Aquat. Microb. Ecol., 67, 1–U119.

[FBV123C14] Hoffman C. S., Winston F. (1987) A 10-minute DNA preparation from yeast efficiently releases autonomous plasmids for transformation of *Escherichia-coli*. Gene, 57, 267–272. 3319781 10.1016/0378-1119(87)90131-4

[FBV123C15] Holl C. M., Montoya J. P. (2005) Interactions between nitrate uptake and nitrogen fixation in continuous cultures of the marine diazotroph *Trichodesmium* (cyanobacteria). J. Phycol., 41, 1178–1183.

[FBV123C16] Hynes A. M., Webb E. A., Doney S. C., Waterbury J. B. (2012) Comparison of cultured *Trichodesmium* (cyanophaecae) with species characterized from the field. J. Phycol., 48, 196–210. 27009664 10.1111/j.1529-8817.2011.01096.x

[FBV123C17] Janson S., Bergman B., Carpenter E. J., Giovannoni S. J., Vergin K. (1999) Genetic analysis of natural populations of the marine diazotrophic cyanobacterium *Trichodesmium*. FEMS Microbiol. Ecol., 30, 57–65.

[FBV123C18] Karl D., Michaels A., Bergman B., Capone D., Carpenter E., Letelier R., Lipschultz F., Paerl H. et al (2002) Dinitrogen fixation in the world's oceans. Biogeochemistry, 57, 47–98.

[FBV123C19] Laroche J., Breitbarth E. (2005) Importance of the diazotrophs as a source of new nitrogen in the ocean. J. Sea Res., 53, 67–91.

[FBV123C20] McCarthy J. J., Carpenter E. J. (1979) *Oscillatoria (Trichodesmium) thiebautii* (cyanophyta) in the central north Atlantic Ocean. J. Phycol., 15, 75–82.

[FBV123C21] Rees A. P., Gilbert J. A., Kelly-Gerreyn B. A. (2009) Nitrogen fixation in the western English Channel (NE Atlantic Ocean). Mar. Ecol. Prog. Ser., 374, 7–12.

[FBV123C22] Rio M. H., Mulet S., Picot N. (2014) Beyond GOCE for the ocean circulation estimate: Synergetic use of altimetry, gravimetry, and *in situ* data provides new insight into geostrophic and Ekman currents. Geophys. Res. Lett., 41, 8918–8925.

[FBV123C23] Sheridan C. C., Steinberg D. K., Kling G. W. (2002) The microbial and metazoan community associated with colonies of *Trichodesmium* spp.: a quantitative survey. J. Plankton Res., 24, 913–922.

[FBV123C24] Smyth T. J., Fishwick J. R., Al-Moosawi L., Cummings D. G., Harris C., Kitidis V., Rees A., Martinez-Vicente V. et al (2010) A broad spatio-temporal view of the Western English Channel observatory. J. Plankton Res., 32, 585–601.

[FBV123C25] UNESCO (ed.) (1968) Monographs on Oceanographic Methodology: Zooplankton Sampling. United Nations, Paris.

[FBV123C26] Xu W., Miller P. I., Quartly G. D., Pingree R. D. (2015) Seasonality and interannual variability of the European Slope Current from 20 years of altimeter data compared with *in situ* measurements. Remote Sens. Environ., 162, 196–207.

