# Supplementary material for: Metabolically active, non-nitrogen fixing, Trichodesmium in UK coastal waters during winter
Source: J Plankton Res. 2016 May 30;38(3):673–8. doi: 10.1093/plankt/fbv123 (PMC4892227; doi:10.1093/plankt/fbv123)
Supplement: Supplementary Data [file supp_38_3_673__index.html]

Metabolically active, non-nitrogen fixing, Trichodesmium in UK coastal waters during winter — Metabolically active, non-nitrogen fixing, Trichodesmium in UK coastal waters during winter — Supplementary Data 

# Metabolically active, non-nitrogen fixing, *Trichodesmium* in UK coastal waters during winter

## Supplementary Data

Supplementary Data

- Supplementary Data - Docx file
