# Supplementary material for: Metabolically active, non-nitrogen fixing, Trichodesmium in UK coastal waters during winter
Source: J Plankton Res. 2016 May 30;38(3):673–8. doi: 10.1093/plankt/fbv123 (PMC4892227; doi:10.1093/plankt/fbv123)
Supplement: Supplementary Data [file supp_fbv123_fbv123supp.docx]

**Supporting information**

**Methods**

**Molecular identification of the *Trichodesmium sp.***

PCR primers were specific to the heterocyst differentiation gene (*hetR*) (primer pair PHI (TGYGCKATTTAYATGACCTA) and PH2 (ATGAANGGTATKCCCCAAGGA) (Lundgren et al. 2005)); the 16S–23S internal transcribed spacer (ITS) (tri16S-1247F (CGTACTACAATGGTTGGG) and tri-23SR (TTCGCTCACCGCTACA) (Orcutt et al. 2002)) and the 16S rRNA gene of Cyanobacteria (CYA359F (GGGGAATYTTCCGCAATGGG) and CYA-781R (GACTACWGGGGTATCTAATCCCW) (Nübel et al. 2007)). PCR reaction mixtures contained 10 ng of DNA, PCR buffer (Qiagen), 3.0 mmol L^-1^ MgCl_2_, 0.1 mmol L^-1^dNTPs, 1 U Taq DNA Polymerase (Qiagen) and 400 μmol L^-1^ of forward and 400 μmol L^-1^ of reverse primer . For the *hetR*gene, PCR conditions were 5 minutes at 95 °C followed by 35 cycles of 95 °C for 30 s, 58 °C for 30 s, 72 °C for 30 s, and a final extension step of 72 °C for 5 minutes. Similar conditions were used for the ITS and 16S rRNA gene PCRs, but the annealing temperature was adjusted to 55 °C and 60 ° C, respectively.

PCR products were cloned into pGEM (Promega) and transformed into *Escherichia coli* JM109 (Promega) according to the manufacturer’s instructions. Ten randomly selected clones from each gene were sequenced by DNA Sequencing Services (Dundee). Nucleotide and amino acid alignments were generated using ClustalW in MEGA 5 (Molecular Evolutionary Genetics Analysis; Tamura et al. 2011). Each of the ten clones for the *hetR*, ITS and 16S rRNA genes showed very few nucleotide mis-matches (>99.5 % similar). Consensus sequences were calculated using BioEdit (Hall et al. 2011). Non-identical nucleotides were identified and compared to the original chromatograms before editing. Sequences obtained in this study have been deposited in GenBank under the following accession numbers: KM269072 (16S rRNA), KM269073 (ITS) and KM269074 (*hetR*).

**Quantitative PCR of *rbcL* mRNA and DNA**

To confirm that the *Trichodesmium* colonies found at L4 were capable of fixing CO_2_, quantitative PCR (qPCR) and reverse transcriptase (RT) qPCR were used to measure the abundance of transcripts of the large subunit of RuBisCO (*rbcL*) relative to gene numbers. On two occasions (29 January and 10 February) colonies collected from the vertical hauls (WP2) net were stored in cool, dark conditions, returned to the laboratory and processed at ambient conditions. Once colonies were picked out, they were placed onto GF/F filters and the filter stored at -80 °C. It is important to acknowledge that collection and storage protocols were not adequate to reflect the true in-situ condition but do allow investigation of the potential for carbon fixation. Both the time elapsed between collection and freezing, and the difference in conditions between transportation and in-situ might alter RNA expression from the natural condition. The filters were then cut in half and each half subjected to a combined DNA and RNA extraction procedure using the AllPrep DNA/RNA mini kit (Qiagen, Manchester, UK). A DNA digestion step was used to remove remaining DNA from the RNA preparations, according to the manufacturer’s instructions. Reverse transcription of total RNA was performed using the QuantiTect Reverse Transcription kit (Qiagen, Manchester, UK). Primers specific to the *rbcL* gene of *T. erythraeum* were designed (Te/rbcLF GTGACCACCTCCACTCAGGT and Te/rbclR ACCACCGAATTGTAAGCAGG) was used to amplify a 254 bp fragment of DNA using 1 µL of DNA or cDNA (22 – 52 ng), 5 µL PCR buffer (Qiagen), 3.0 mmol L^-1^ MgCL_2_, 0.2 mmol L^-1^ dNTPS, I U Taq DNA polymerase (Qiagen) and 200 μmol L^-1^ of forward and 400 μmol L^-1^ of reverse primer and the following amplification conditions: 5 min at 95 °C followed by 35 cycles of 95 °C for 30 s, 52 °C for 45 sec and 72 °C for 45 sec, with a final extension of 5 min at 72 °C. The resultant PCR product was then cloned and sequenced as described above, and identified by comparison to published *rbcL* genes in the Genbank database. Eight clones were sequenced from each time-point: all contained the same gene sequence which had 92 % identity to the *T. erythraeum rbcL* gene (accession number KU221396). To *rbcL* mRNA and DNA quantification, an ABI 7000 sequence detection system (Applied Biosystems, Foster City, USA) and QuantiFast SYBR Green PCR Kit (Qiagen) was used. The 20 µl reaction mixture contained 10 µl of Master Mix, 300 nM each of Te/rbcL primer and 1 μl of DNA or cDNA. PCR conditions were 5 min at 95 °C followed by 40 cycles of 95 °C for 15 s, 52 °C for 30 sec and 72 °C for 45 sec and melting curve analysis. Assays contained a standard curve containing 10^2^ to 10^8^ amplicons µl ^-1^ cDNA or DNA. DNA standard curves were constructed using cloned sequences. For RT qPCR, standard curves were produced from cDNA following prior *in vitro* transcription of cloned sequences using the Ampliscribe T7 Flash kit (Epicentre) following methodologies described by Smith et al. (2006). Absence of DNA within the RNA preparations was confirmed by absence of a PCR product using the above reagents and conditions with 1 µL or RNA and any amplification tracked using RT-PCR. Gene and transcript numbers were quantified *via* comparison to standard curves using the ABI Prism 7000 detection software. Automatic analysis settings were used to determine the threshold cycle (C_T_) values and baselines settings. The no-template controls were below the threshold in all experiments. For each standard curve, the slope, *y* intercept, co-efficient of determination (*r^2^*) and the efficiency of amplification determined as follows: cDNA reactions: *r^2^* = 0.998, *y* intercept = 39.09, *E* (amplification efficiency) = 87.34 %, DNA reactions: *r^2^* = 0.993, *y* intercept = 40.15, *E*  = 97.96 %,

Hall, T. (2011). BioEdit: An important software for molecular biology. GERF Bull. Biosci, 2, 60-61.

Lundgren, P., Janson, S., Jonasson, S., Singer, A., Bergman, B. (2005) Unveiling of novel radiations within Trichodesmium cluster by hetR gene sequence analysis. Appl. Environ. Microbiol. 71, 190–196.

Nübel, U., Garcia-Pichel, F., Muyzer, G. (1997) PCR primers to amplify 16S rRNA genes from cyanobacteria. *Appl. Environ. Microbiol.* **63**, 3327–3332.

Orcutt, K.M., Rasmussen, U., Webb, E.A., Waterbury, J.B., Gundersen, K., Bergman, B. (2002) Characterization of *Trichodesmium* spp. by genetic techniques. *Appl. Environ. Microbiol.* **68**, 2236–2245.

Smith, C.J., Nedwell, D.B., Dong, L.F., Osborn, A.M. (2006) Evaluation of quantitative polymerase chain reaction-based approaches for determining gene copy and gene transcript numbers in environmental samples. *Environ Microbiol* 8: 804-815.

Tamura, K., Peterson, D., Peterson, N., Stecher, G., Nei, M., Kumar, S. (2011) MEGA5: molecular evolutionary genetics analysis using maximum likelihood, evolutionary distance, and maximum parsimony methods. *Mol. Biol. Evol.* **28**, 2731-2739.
